# Inflammation, the kynurenines, and mucosal injury during human experimental enterotoxigenic *Escherichia coli* infection

**DOI:** 10.1007/s00430-024-00786-z

**Published:** 2024-03-02

**Authors:** Sehee Rim, Oda Barth Vedøy, Ingeborg Brønstad, Adrian McCann, Klaus Meyer, Hans Steinsland, Kurt Hanevik

**Affiliations:** 1 Department of Clinical Science, Faculty of Medicine, University of Bergen, Bergen, Norway; 2 National Centre for Ultrasound in Gastroenterology, Haukeland University Hospital, Bergen, Norway; 3 Department of Medicine, Haukeland University Hospital, Bergen, Norway; 4 Bevital AS, Bergen, Norway; 5 Department of Global Public Health and Primary Care, Faculty of Medicine, Centre for Intervention Science in Maternal and Child Health, Centre for International Health, University of Bergen, Bergen, Norway; 6 Department of Biomedicine, Faculty of Medicine, University of Bergen, Bergen, Norway; 7 Department of Medicine, National Center for Tropical Infectious Diseases, Haukeland University Hospital, Bergen, Norway

**Keywords:** Experimental infection, Calprotectin, Longitudinal, Truncation, Tryptophan, Indoleamine 2,3-dioxygenase

## Abstract

Enterotoxigenic *Escherichia coli* (ETEC) is an important cause of diarrhea in children and travelers, especially in low- and middle-income countries. ETEC is a non-invasive gut pathogen colonizing the small intestinal wall before secreting diarrhea-inducing enterotoxins. We sought to investigate the impact of ETEC infection on local and systemic host defenses by examining plasma markers of inflammation and mucosal injury as well as kynurenine pathway metabolites. Plasma samples from 21 volunteers experimentally infected with ETEC were collected before and 1, 2, 3, and 7 days after ingesting the ETEC dose, and grouped based on the level of intestinal ETEC proliferation: 14 volunteers experienced substantial proliferation (SP) and 7 had low proliferation (LP). Plasma markers of inflammation, kynurenine pathway metabolites, and related cofactors (vitamins B2 and B6) were quantified using targeted mass spectrometry, whereas ELISA was used to quantify the mucosal injury markers, regenerating islet-derived protein 3A (Reg3a), and intestinal fatty acid-binding protein 2 (iFABP). We observed increased concentrations of plasma C-reactive protein (CRP), serum amyloid A (SAA), neopterin, kynurenine/tryptophan ratio (KTR), and Reg3a in the SP group following dose ingestion. Vitamin B6 forms, pyridoxal 5'-phosphate and pyridoxal, decreased over time in the SP group. CRP, SAA, and pyridoxic acid ratio correlated with ETEC proliferation levels. The changes following experimental ETEC infection indicate that ETEC, despite causing a non-invasive infection, induces systemic inflammation and mucosal injury when proliferating substantially, even in cases without diarrhea. It is conceivable that ETEC infections, especially when repeated, contribute to negative health impacts on children in ETEC endemic areas.

**Supplementary Information:**

The online version contains supplementary material available at 10.1007/s00430-024-00786-z.

## Introduction

Infection with enterotoxigenic *Escherichia coli* (ETEC) causes an estimated 75 million diarrheal episodes and 50,000 deaths annually, mostly in children under the age of 5 years living in low- and middle-income countries (LMICs) as well as among travelers and military personnel deployed in these countries [1, 2]. ETEC colonizes the small intestinal cell wall non-invasively and releases one or both protein enterotoxins, heat-stable toxin (ST), and heat-labile toxin (LT), which induce diarrhea [3]. Repeated episodes of acute or persistent diarrhea in early childhood, followed by ETEC infection can result in malnutrition and stunting [4]. Despite considerable efforts to develop vaccines against ETEC to prevent diarrheal diseases, effective vaccines have not yet been licensed [5]. By studying the markers after ETEC infection, immune responses can be verified to examine the efficacy of vaccine candidates and help understand the pathogenicity of ETEC.

The gastrointestinal tract is well protected against enteric pathogens, but enteric pathogens can break through these defenses to colonize or invade the cell wall and cause gastrointestinal diseases [6]. Although ETEC is a non-invasive pathogen, infection with ETEC is assumed to induce mild systemic inflammation [7], which is supported by recent studies that have identified increased concentrations of fecal inflammation markers in infected adults and infants [2, 8].

Gazi et al*.* compared the levels of different fecal markers of inflammation in ETEC-positive and ETEC-negative children aged 12–18 months. They found that ETEC infections led to increased calprotectin levels and reduced neopterin levels in stools, while myeloperoxidase (MPO) levels did not seem to change [8]. Brubaker et al*.* found increased serum concentrations of intestinal fatty acid-binding protein (iFABP), IL-17A, and IFN-γ, as well as fecal MPO in volunteers who developed diarrhea after experimental infection with human ETEC [2]. Experimental ETEC infections, including a study investigating the same cohort as in this study, have also been shown to induce systemic cellular immune responses, as identified by increased levels of activated innate and adaptive immune cells [9, 10]. These studies support the hypothesis that ETEC infections lead to activation of gastrointestinal and/or systemic inflammation and immune responses.

Several plasma markers are considered suitable for identifying the type and magnitude of acute-phase responses to bacterial infections in humans [11]. C-reactive protein (CRP) is the most commonly used clinical marker for assessing the grade of infection, tissue damage, and inflammation [12]. Serum amyloid A (SAA) isoforms represent a major type of apolipoprotein, the plasma concentration of which can increase up to 1000-fold during inflammation, particularly in cases of infections caused by Gram-negative bacteria [13]. SAA has various isoforms and truncation patterns, with their roles potentially specific to unique pathways during infection and inflammation; however, their roles are not yet well understood. Plasma neopterin concentration is a useful marker for early cellular immune responses because it reflects the levels of activated macrophages and dendritic cells that are activated by interferon gamma (IFN-γ) from T lymphocytes. [14, 15]. Calprotectin belongs to the S100 family of Ca-binding proteins and represents different heterocomplexes of S100A8, S100A9, and an isoform of truncated S100A9. Serum calprotectin levels increase during systemic inflammation [16], especially during inflammation in the gut, but are less sensitive than fecal calprotectin as a diagnostic marker [17]. PAr (pyridoxal activation ratio) index is another inflammation marker derived from the B6 vitamers, defined as 4-pyridoxic acid (PA) divided by the sum of pyridoxal 5ʹ-phosphate (PLP) and pyridoxal (PL)), which has recently been found associated mainly to chronic inflammation [18].

Host immune responses to infection can also be investigated by monitoring the changes in the levels of circulating kynurenines, which represent different types of tryptophan metabolites. Another useful measure is the kynurenine/tryptophan ratio (KTR), which reflects the pro- and anti-inflammatory activities of indoleamine 2,3-dioxygenase (IDO) [19]. Other kynurenines, like 3'-hydroxykynurenine (HK), quinolinic acid (QA), and picolinic acid (Pic) can also be used to monitor the activation or modulation of inflammatory responses [20].

Some markers of gut-specific mucosal injury can be measured in plasma. Regenerating islet-derived protein 3A (Reg3a) is a bactericidal, gut-specific protein that is often secreted in response to inflammation during the acute phase of infection to protect the gut epithelium [21, 22]. Intestinal fatty acid-binding protein 2 (iFABP) is another small intestine-specific protein that is involved in the metabolism of long-chain fatty acids and can be used as a marker for gut injury [23].

Understanding human inflammatory responses to ETEC infections may help us identify useful measures to counter the detrimental effects of ETEC infections and guide vaccine development by validating inflammation and immune responses. In this study, using proliferation data and targeted metabolomics data from plasma samples collected from humans experimentally infected with ETEC [24], we aimed to investigate mucosal injury and inflammatory responses to experimental ETEC infection.

## Materials and methods

### Experimental infection studies

The present study was based on analyses of specimens and data from two experimental ETEC infection studies performed at Haukeland University Hospital, Bergen, Norway, from 2016 to 2018 [25, 26]. In these studies, 21 volunteers were infected with ETEC strain TW10722 and 9 with strain TW11681, and in this study, we present specimens and data from the last 12 infected with TW10722 and all 9 infected with TW11681. Both strains produce the human variant of the heat-stable toxin (STh), but not the LT. TW11681 (O19:H45) produces the colonization factors CFA/I and CS21, while TW10722 (O115:H5) produces CS5 and CS6.

All volunteers received the same hospital food throughout their hospitalization, resulting in minimal dietary variation. Pre-existing gastrointestinal diseases and recent travel to endemic countries within the past year served as exclusion criteria for study participation and were rigorously screened.

None of the volunteers required oral or intravenous fluids during the night, and they had fasted for at least 7 h before blood specimens were collected in the morning immediately before and 1, 2, 3, and 7 days after they had ingested the ETEC dose. Blood was collected by venipuncture into pre-cooled ethylenediaminetetraacetic acid tubes, which were kept on ice until centrifuged at 1800×*g* for 10 min at 4 °C. Within 30 min of venipuncture, the resulting plasma was stored at – 80 °C until further analysis.

### Ethics

Written informed consent was obtained from all volunteers. The study was approved by the Regional Committee for Medical and Health Research Ethics, Health Region West (Project ID: 2014–826) and registered at ClinicalTrials.gov (Project ID: NCT02870751).

### Biomarker analyses

#### ETEC proliferation level determination

We used the maximum observed proportion of ETEC DNA relative to total DNA in volunteer stool specimens as a measure of ETEC proliferation, as described by Vedøy et al*.* [24, 27]. In brief, DNA from up to three stool samples by each volunteer each day following experimental infection, was extracted and purified. Total DNA amount in each extract was measured with a fluorescent dye assay, and ETEC DNA was quantified by using quantitative PCR (qPCR) in assays targeting the O19 (for TW11681) or O115 (for TW10722) variants of the O-antigen polymerase gene (*wzy*). *wzy-*based PCR assays can be made highly specific for any of a wide range of *E. coli* types, where both O19- and O115-producing *E. coli* are rarely isolated from humans [28] and O-antigen polymerase gene is only found in a single copy on the *E. coli* chromosome with the gene sequences are unique for each O antigen [29]. In addition, qPCR analyses of the volunteers' first stools gave no clear indications that any of the volunteers were harboring such *E. coli* prior to study start. qPCR is proven to be a trusted method to quantitate gut bacterial proliferation [30, 31].

All volunteers with a maximum concentration of ≥ 0.99% TW10722 DNA also developed diarrhea (Table 1) [24]. Therefore, we considered volunteers who had ≥ 0.99% TW10722 or TW11681 DNA concentrations to have substantial proliferation (SP), whereas those who always had < 0.99% were considered to have low proliferation (LP).

**Table 1:** Volunteer characteristics for the age, proliferation level, and time to diarrhea according to the ETEC proliferation group. Data shown as median (range) values

	Substantial proliferation (SP; *N* = 14)	Low proliferation (LP; *N* = 7)
Age, years (range)	23 (22–28)	23 (20–28)
Sex, females, N (%)	12 (85%)	7 (100%)
Challenge strain, N		
TW11681	6	3
TW10722	8	4
Doses given (range)		
TW11681	10^6^–10^9^	10^6^–10^9^
TW10722	10^9^–10^10^	10^9^–10^10^
Proliferation level, median (range)	4.22 (0.99–10.79)	0.26 (0.01–0.62)
Volunteers with diarrhea, *N*	10	0
Time to diarrhea, h, median (range)	25.5 (18–80)	–

#### Intestinal mucosal injury markers

Plasma concentrations of regenerating islet-derived protein 3 alpha (Reg3A) were estimated using the Human Reg3A DuoSet ELISA kit (Catalog # DY5940-05; R&D Systems, Minneapolis, MN, USA) (Table 2). Intestinal fatty acid-binding protein 2 (iFABP) was quantified using the Quantikine® ELISA kit (Human FABP2/IFABP, catalog number DFBP20) from R&D Systems. The ELISAs were performed according to the manufacturer’s instructions. Plasma samples were diluted 50- to 300-fold before the analysis to fit the calibration curve. The optical density was measured using a SPECTRAmax microplate reader (Molecular Devices, Sunnyvale, CA, USA) set to 450 nm with a correction wavelength of 540 nm. Plasma concentrations were determined using the four-parameter algorithm in SoftMaxPro version 7.1.

**Table 2:** Overview plasma markers analyzed in the present study

Category	Metabolite	**Abbreviation**	Concentration units
Inflammation	C-reactive protein	**CRP**	µg/mL
	Neopterin	**Neopt**	nmol//L
	Serum amyloid A (total)	**SAAt**	µg/mL
	Serum amyloid A isoforms 1.1, 1.1dr, 1.1drs, 1.2, 1.2dr, 1.2drs, 1.3, 1.3drs, 2.1, 2.1dr, 2.1drs, 2.2, 2.2dr, 2.2drs	**SAAvar**	μg/mL
	Calprotectin (total)	**S100At**	µg/mL
	isoform S100A8	**S100A8**	µg/mL
	isoform S100A9	**S100A9**	µg/mL
Kynurenines	Kynurenine/tryptophan ratio	**KTR**	
	Kynurenine	**Kyn**	nmol/L
	Tryptophan	**Trp**	µmol/L
	Kynurenic acid	**KA**	nmol/L
	Xanthurenic acid	**XA**	nmol/L
	Anthranilic acid	**AA**	nmol/L
	3-Hydroxyanthranilic acid	**HAA**	nmol/L
	Picolinic acid	**Pic**	nmol/L
	3'-Hydroxykynurenine	**HK**	nmol/L
	Quinolinic acid	**QA**	nmol/L
Vitamin B1	Thiamine	**Thi**	nmol/L
	Thiamine monophosphate	**TMP**	nmol//L
Vitamin B2	Riboflavin	**Ribo**	nmol//L
	Flavin mononucleotide	**FMN**	nmol//L
Vitamin B3	Nicotinic acid	**NA**	nmol/L
	Nicotinamide	**NAM**	nmol/L
	N1-methylnicotinamide	**mNAM**	nmol/L
Vitamin B6	Pyridoxal activation ratio; PA/(PLP + PL)	**PAr**	nmol//L
	Pyridoxal 5'-phosphate	**PLP**	nmol//L
	Pyridoxal	**PL**	nmol//L
	4-Pyridoxic acid	**PA**	nmol//L
Intestinal mucosa injury	Intestinal fatty acid-binding protein-2	**iFABP**	pg/mL
	Regenerating islet-derived protein III-alpha	**Reg3a**	pg/mL

#### Inflammation and immune activation markers

The plasma concentrations of the inflammation markers, including CRP, SAAt, the Serum amyloid A isoforms SAA1.1–1.3 and SAA2.1–2.2 (SAAvar), and the total calprotectin (S100At) and its isoforms S100A8 and S100A9 were quantified by Bevital AS, Bergen, Norway (www.bevital.no) using matrix-assisted laser desorption/ionization time-of-flight mass spectrometry (MALDI–TOF-MS) [32–34]. Within- and between-day coefficients of variation in the estimated concentrations ranged from 4 to 7% for inflammation markers, except for calprotectin, and 4–10% for calprotectin and its isoforms.

Nine metabolites of the kynurenine pathway, which is closely linked to inflammatory and immune activation processes, were analyzed using liquid chromatography-tandem mass spectrometry (LC/MS–MS). The metabolites include tryptophan (Trp), kynurenine (Kyn), 3-hydroxykynurenine (HK), kynurenic acid (KA), xanthurenic acid (XA), anthranilic acid (AA), 3-hydroxyanthranilic acid (HAA), picolinic acid (Pic), and quinolinic acid (QA) [35, 36].

Neopterin (Neopt), a marker for immune system activation, pyridoxal 5ʹ-phosphate (PLP), a marker for vitamin B6 status, and riboflavin, a marker for vitamin B2 status, were also analyzed using LC/MS–MS [35, 36]. Within- and between-day coefficients of variation in the estimated concentrations of the metabolites ranged from 2 to 10% for the nine metabolites in the kynurenine pathway, and 2–17% for the latter three metabolites.

Finally, indexes defined as the ratios of the two metabolites were calculated. The kynurenine-to-tryptophan ratio (KTR) is used as an indicator of inflammation and IDO activation and was calculated by dividing the serum concentration of Kyn (in nmol/L) by the serum concentration of Trp (in µmol/L) [19, 37]. The PAr index is the ratio of 4-pyridoxic acid (PA) to the sum of PLP and pyridoxal (PL) concentrations [18]. PAr is used to monitor changes in vitamin B6 catabolism, which increases during inflammation, is usually observed in chronic inflammation, and is only slightly influenced by vitamin B6 intake [38].

### Statistics

To identify changes in marker concentrations within the SP and LP groups before dose ingestion (baseline) and during follow-up, we used the Friedman test, where we also compensated for multiple comparisons using the Bonferroni correction. For markers that displayed changes in concentrations during follow-up, we used the Conover–Iman post hoc test to identify days with pairwise differences in marker concentrations from the baseline. All *p* values in this text were noted from the Conover–Iman test, not the *p* values from the Friedman test or after Bonferroni correction. The Mann–Whitney *U* test was used to discern differences in marker concentrations between volunteer groups with substantial and low proliferation between the two time points. Correlations between marker concentrations and maximum DNA proliferation levels in stools were evaluated using Spearman’s tests to examine monotonical correlations. All statistical tests were performed using Python package *statsmodels* [39]. Line graphs and regression plots were created using the Python packages *matplotlib*, *seaborn*, and *bioinfokit* [40–42].

For further analysis of SAA isoforms and their truncation patterns into SAAdr and SAAdrs isoforms during the follow-up period, we performed the Friedman test over the days, corrected by Bonferroni and on to the Conover–Iman test for the ratio of original and truncated isoforms, measured as proportions of each isoform concentration divided by SAAt for each day and volunteer. A radar plot for the truncation patterns was created using Microsoft Excel.

## Results

The 21 volunteers included in this study were young adults (median age, 23 years), consisting of 19 females and 2 males (Table 1). They were grouped according to the maximum observed stool ETEC concentration following the experimental infection, with the 14 volunteers, who had their maximum values higher than 0.99% (median 4.2%, range 0.99–10.79%), representing the substantial ETEC proliferation (SP) group, and the 7 volunteers with their maximum values less than 0.99% (median 0.26%, range 0.01–0.62%), representing the low proliferation (LP) group [24, 27].

In the SP group, the median time from oral inoculation to peak stool proliferation levels was 3 days, whereas discernible peaks were not observed in the LP group. There were no differences in age or sex distribution between the two groups (Table 1).

Eight volunteers reported various types of allergy, mostly pollen allergies, including sensitivities to dust, mites, timothy grass, sports tape, and penicillin. We did not see any clear association between having known allergies and experiencing higher ETEC proliferation.

In the SP group, there were ten volunteers who developed diarrhea and four volunteers who did not, with a starting time point for diarrhea ranging from 18 to 80 h (median: 25.5 h). None of the volunteers in the LP group developed diarrhea. There were no differences in the baseline levels between the SP and LP groups for any of the investigated markers.

### Markers of inflammation and immune activation

**Fig. 1:**
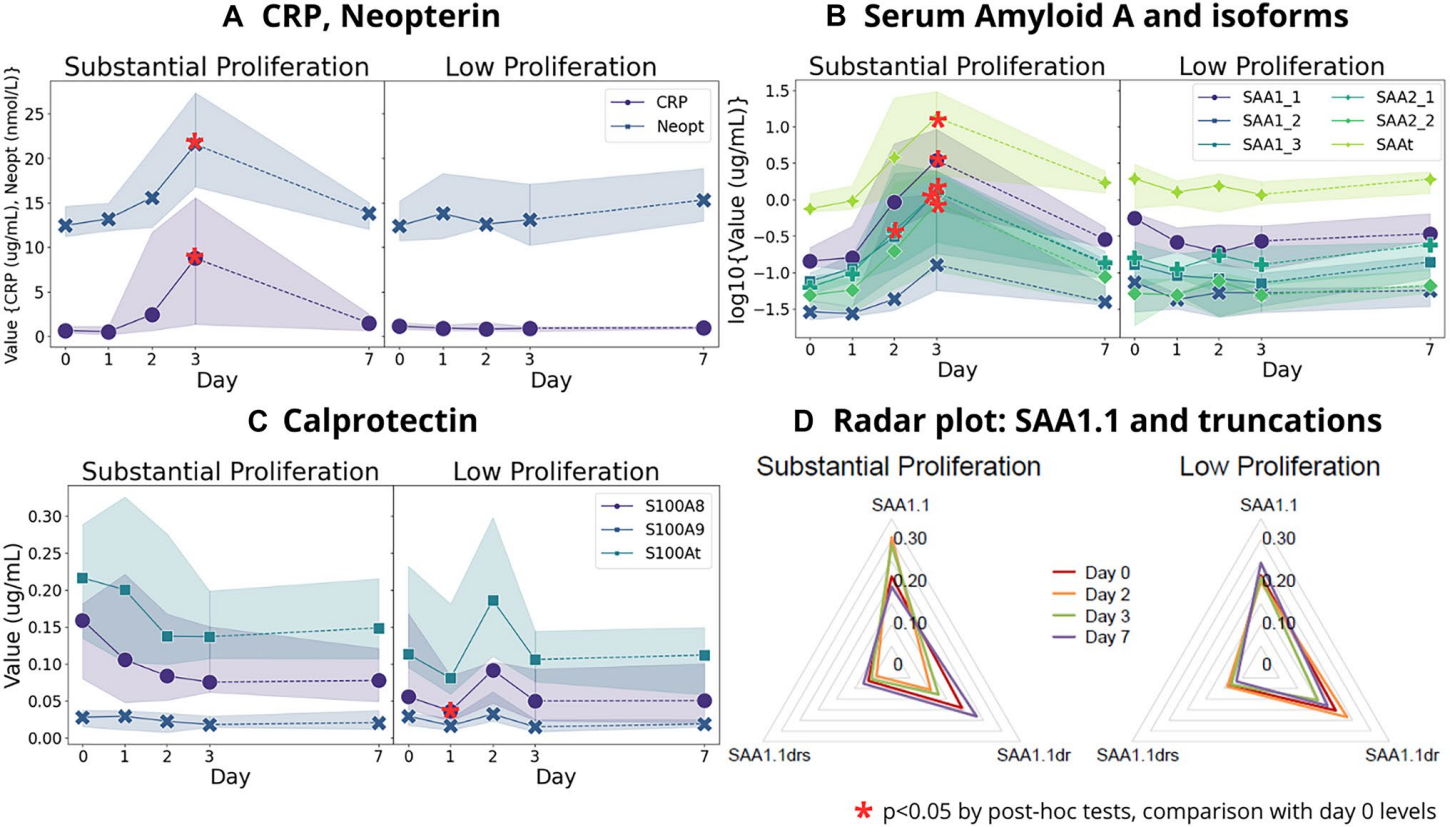
**A**, **B**, **C** represent line graphs of median values of plasma levels of inflammation markers in the substantial proliferation group and low proliferation group on each day, with bands ranging from the 25th percentile to 75th percentile. For SAA isoforms, shown in B, values are represented in log values. **D** shows a radar plot illustrating the kinetics of the SAA1.1 isoform ratios and its truncated variant patterns over four time points. Values with asterisks represent the significant change from the baseline (day 0) to the corresponding day as results of post hoc tests, with the detailed values specified in the Supplementary Tables 1, 2–1 and 2–2

Several inflammatory markers were increased in the SP group during ETEC infection, in contrast to almost no change in the LP group (Fig. 1). The markers with changes from baseline included C-reactive protein (CRP), neopterin, and serum amyloid A (SAA). These values increased from baseline in the SP group, with the highest levels on day 3 after dose ingestion, returning to baseline levels by day 7 (Fig. 1A, B). The increases were significant after multiple test corrections and post hoc tests for CRP (*p* = 0.041), neopterin (*p* < 0.001), and SAAt (*p* < 0.001). We also observed increases in several variants of SAA, including SAA1.1 (*p* = 0.004), SAA1.3 (*p* = 0.009), and SAA2.1 (*p* < 0.001), which exhibited their highest values on day 2 or 3 of the examined time points in the SP group (Fig. 1B).

Investigation of SAA isoform ratios, defined as each SAA isoform divided by the total SAA for each day and volunteer, revealed an altered truncation pattern in SAA1.1 over the infection phase. While the SAA1.1 ratio was the highest on day 2, its truncated forms, the SAA1.1 dr ratio, and the SAA1.1 drs ratio decreased on days 2 and 3, returning to baseline on day 7 (Fig. 1D). The Conover–Iman test revealed differences in the SP group for the SAA1.1 ratio between days 2 and 7, SAA1.1 dr ratio between days 0 and 3, and SAA1.1 drs ratio between days 3 and 7. None of the other SAA isoform ratios showed this trend (Supplementary Fig. 1).

The LP group did not show any increase in CRP or SAAt levels. When comparing marker levels between groups on each day, differences reached significance on day 3 for CRP (*p* = 0.038), SAAt (*p* = 0.006), and all SAA variants except for SAA1.2 (Fig. 1A, B).

Total plasma calprotectin (S100At) showed a significant, but marginal decrease in the LP group, from baseline to day 1 (*p* = 0.012) and day 3 (*p* = 0.042), and similar decreases for subvariant S100A9 in both groups (Fig. 1C).

### The kynurenines and vitamin B, cofactors of the kynurenine pathway

**Fig. 2:**
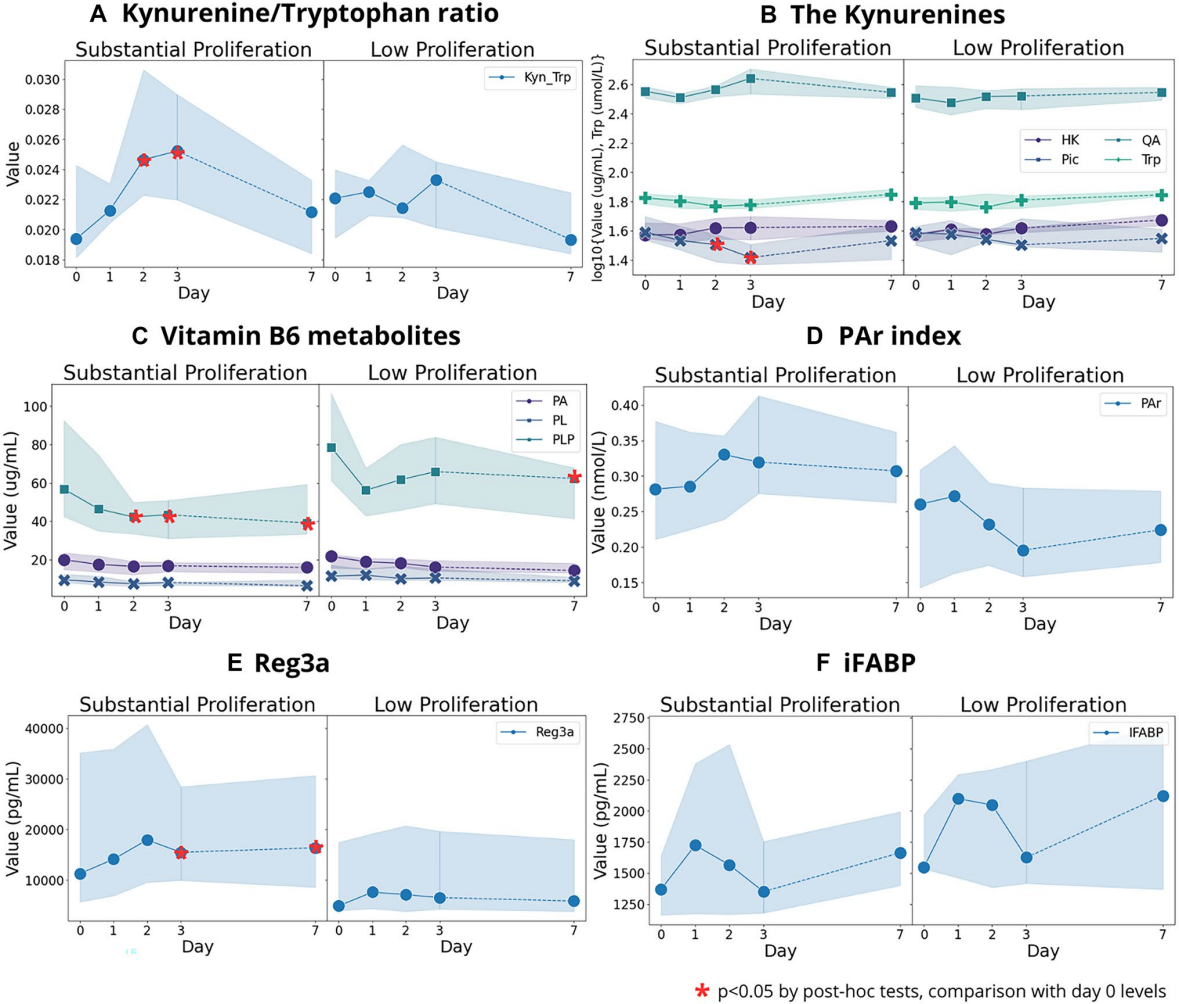
Line graphs show the median values of plasma levels of metabolites of kynurenine and vitamin B6 together with mucosal injury markers in the SP group and LP group on each day, with bands ranging from the 25th percentile to 75th percentile. For the kynurenines, we took only significant markers for simplicity and values represented in log values. Values with asterisks represent the significant change from the baseline (day 0) to the corresponding day as results of post hoc tests, with the detailed values specified in the supplementary Table 1, 2–1 and 2–2. *HK* 3ʹ-hydroxycotinine, *Pic* picolinic acid, *QA* quinolinic acid, *Trp* tryptophan, *PA* 4-pyridoxic acid, *PL* pyridoxal, *PLP* pyridoxal 5ʹ-phosphate, *PAr index*, PA: (PLP + PL)

KTR increased from baseline to days 2 (*p* = 0.025) and 3 (*p* = 0.018) (Fig. 2A) in the SP group. Interestingly, Pic decreased until day 3 after dose ingestion in both groups, but only significantly in the SP group on days 2 (*p* = 0.048) and 3 (*p* = 0.0014), before returning to baseline levels (Fig. 2B). Quinolinic acid (QA) and 3'-hydroxykynurenine (HK) did not show any change from baseline, with stable values in both the SP and LP groups.

Regarding cofactors of the kynurenine pathway, we found that plasma riboflavin (B2 vitamer) levels fluctuated in both groups without changes. However, the vitamin B6 metabolite pyridoxal 5ʹ-phosphate (PLP) gradually decreased from baseline to days 2 (*p* = 0.006), 3 (*p* < 0.001), and 7 (*p* = 0.015) in the SP group (Fig. 2C). A sharp, insignificant drop in PLP was also observed in the LP group, but the levels quickly rebounded. Comparing the two groups, we found that the levels of PL (day 2, *p* = 0.007; day 3, *p* = 0.030) and PLP (day 3, *p* = 0.031) were lower in the SP group. The pyridoxic acid ratio (PAr) index, the result of PA:(PLP + PL), did not show any change from baseline (Fig. 2D), but was higher in the SP group on day 3 (*p* = 0.025). Vitamins B1 and B3 did not show notable changes.

### Markers of mucosal injury

Reg3a levels were highly variable between individuals, but increased in the SP group and reached higher levels on day 3 (*p* = 0.002) and remained elevated on day 7 (*p* = 0.025) after dose ingestion (Fig. 2E). In addition, iFABP levels were highly variable inter-individually, but did not show any changes, although the marker showed an upward trend at day 1 in both groups (Fig. 2F).

### Association between plasma markers

Some markers correlated with one another, especially between most inflammation markers. Additionally, Reg3a was positively correlated with calprotectin on days 0 (*p* = 0.011) and 3 (*p* = 0.009) (Supplementary Fig. 2). Some metabolites of the kynurenine pathway, anthranilic acid (AA) on day 2, HK and QA on days 2 and 3, correlated well with KTR as expected, but also with other inflammation markers, including PAr index, neopterin, CRP, and SAAt (Supplementary Table 3).

### Association between plasma markers and ETEC proliferation

Maximum stool ETEC DNA concentrations, as a measure of ETEC proliferation, weakly correlated with some inflammatory markers on day 3, namely CRP (*γ* = 0.45, *p* = 0.04), SAAt (*γ* = 0.48, *p* = 0.027), and PAr (*γ* = 0.49, *p* = 0.025) (Supplementary Fig. 3). We also found weak positive correlations between the maximum ETEC proliferation level and thiamine (*γ* = 0.47, *p* = 0.033) and negative correlations with PLP (*γ* = – 0.53, *p* = 0.013), PL (*γ* = – 0.47, *p* = 0.03), and iFABP (*γ* = – 0.45, *p* = 0.04) levels on day 3. The regression plots for these correlations are shown in Supplementary Fig. 4.

## Discussion

We investigated plasma biomarkers of inflammation, immune activation, kynurenines, and mucosal injury in relation to ETEC proliferation during human experimental ETEC infection. CRP, neopterin, and serum amyloid A levels increased substantially and reached maximum levels 3 days after dose ingestion, while no clear changes were seen in these levels for any of the LP volunteers. Plasma calprotectin levels decreased from baseline in the LP group. Among the kynurenines, KTR increased until day 3 and Pic decreased in the SP group. B6 vitamers, cofactors of kynurenines, PLP, and PL were lower in the SP group on day 3. The mucosal injury marker Reg3a increased in circulation until day 2, particularly in the SP group.

### Markers of inflammation and immune activation

CRP levels increased in the SP group on day 3 after dose ingestion, indicating a mild systemic inflammatory condition induced by ETEC. A rise in CRP usually reaches the max level within 48 h after microbe invasion and tissue damage, and this resonates well with the peak point for CRP since the median time to first symptom debut was 25.5 h for volunteers with the diarrheal symptom in the SP group. CRP creates a pro-inflammatory environment by activating complement, phagocytosis, nitrogen oxide (NO), and the production of pro-inflammatory cytokines, and eliciting adaptive, humoral immune reactions [43].

SAA is another major acute-phase reactant, and this pro-inflammatory protein exists in several isoforms [44]. In our study, the levels of total SAA and most isoforms increased until day 3 in the SP group. SAA, especially SAA1, contributes to innate immunity by opsonization, especially against Gram-negative bacteria, including *Escherichia coli* and *Pseudomonas aeruginosa*, via phagocytosis by IL-10- and TNF-α-stimulated macrophages [45]. Therefore, it can be argued that SAA plays an active role in protective innate immunity during ETEC infection. In addition, SAA contributes by inducing cytokines, activating cellular surface receptors such as Toll-like receptors 2 and 4, CD36 and stimulating the inflammasome cascade [46].

The truncation pattern of SAA1.1 was altered during the infection phase, where relatively lower levels of SAA1.1 were found as truncated variant on day 2 and 3, and normalizing by day 7. This finding of cessation of SAA1.1 truncation during the early infection phase indicates a potential role of SAA1.1 in early immunity; however, their exact roles and truncation are yet to be studied. Truncations are mainly the result of alternative splicing and cleavage and may also be associated with changes in the expression of truncated forms, protease activities, or its potential function in opsonization.

In the present study, plasma neopterin levels were increased on day 3 in the SP group. A similar increase has been previously reported in individuals infected with *Shigella* [47], an invasive pathogen that normally causes more severe infections than ETEC. In contrast, previous studies found lower fecal neopterin levels in infants positive for ETEC and other gut pathogens in infants in LMIC [8, 48]. This discrepancy can be explained by neopterin functioning as an immunoattractant in circulation rather than secretion into the gastrointestinal tract or biological differences in infants in LMIC.

Calprotectin levels showed a slow decrease until day 3 in the LP group. The S100A8-S100A9 complex functions as an antimicrobial by chelating Zn, Fe, and Mn ions, which are essential for bacterial growth, as observed in an in vivo experiment with *E. coli* [49]. S100A9 itself is also known to enhance phagocytosis by neutrophil granulocytes, as reported for *E. coli *in vivo [50]. In Bangladesh, higher fecal calprotectin levels have been observed in ETEC-positive infants [8]. In patients with inflammatory bowel diseases (IBD) with high fecal calprotectin, fecal and plasma calprotectin usually showed positive associations [51].

### The kynurenines and vitamin B6

KTR showed a sharp increase until day 3 in the SP group. Increased KTR has been highlighted as a candidate for prognostic biomarker for the severity of environmental enteric dysfunction (EED), tested in infants in low-income countries [52]. It is worth noting that in pigs experimentally infected with ETEC, supplementary tryptophan did not have any effect on CRP-level after infection, diarrheal disease, or ETEC shedding, but increased serum serotonin levels, which in turn increased post-weaning performance [53].

An increase in KTR is a result of increased IDO activity, as IDO transforms tryptophan to kynurenine [19], and increased IDO activity can be both beneficial and detrimental for host immunity [54]. IDO protects the host by two mechanisms: first, by degrading tryptophan, a crucial amino acid for the survival of many microorganisms, including *E. coli* [55], and second, by producing kynurenine, which has a bactericidal function. However, IDO activity limits host immunity by inducing the proliferation of regulatory T (Treg) cells, blocking the conversion of Tregs into T effector cells, and increasing T cell apoptosis [20, 54]. Interestingly, uropathogenic *E. coli* (UPEC) is known to induce IDO in the host environment to utilize these immunosuppressive effects for survival and colonization in the host [55].

Picolinic acid (Pic) levels decreased in both groups until day 3, with a more pronounced decrease in the SP group. Pic has been reported to exert antiviral and antimycobacterial effects during HIV and *Mycobacterium avium* infections, respectively [56, 57]. Similar to discussions on the KTR, Pic has been shown to have both beneficial and non-beneficial immunological properties during infection. Pic can activate macrophages [57, 58], but can also inhibit the proliferation of CD4^+^ T cells [59].

Among vitamin B6 forms, PLP, the prevailing bioavailable form in plasma, decreased over time and was negatively correlated with ETEC proliferation. These findings imply exhaustion or reduced uptake of vitamin B6. Thus, these results imply that vitamin B6 could be a beneficial supplement for ETEC infection. Vitamin B6 has antibacterial functions, and its deficiency can negatively affect antibody production and IL-2 and T cell proliferation [60]. Vitamin B6 is a cofactor in the kynurenine pathway, which is associated with ETEC infection [20]. It is also worth noticing that pyridoxine is damaged in autoclaved formula for infants [61], stressing the value of breastfeeding and/or proper administration of vitamin B6 in other forms to young children residing in endemic areas.

The PAr index is known to increase in a chronic inflammatory environment, especially in cardiovascular diseases [18, 62], but no changes were observed in the present study. This may indicate that vitamin B6 catabolism is not significantly affected during acute, temporary, or mild infection.

### Mucosal injury and colonization

Reg3a levels increased to day 3 in the SP group. In porcine diarrheal infection with *Lactobacillus casei*, murine Reg3a promotes intestinal cell proliferation and reduces the bacterial load [63], thus indicating the possibility that Reg3a also plays a role in intestinal host immunity in ETEC infection. In addition, Gazi et al*.* found higher levels of fecal Reg1b, the same family as Reg3a, in enteroaggregative *E. coli* (EAEC)-positive infants but not in ETEC-positive infants [8]. Reg1b is a newly suggested gut-specific marker related to cell proliferation and regeneration and has been suggested as a prognostic marker for stunting among infants in Bangladesh and Peru [64]. This suggests that plasma Reg3a may also be a biomarker of intestinal injury. Elevated systemic inflammatory markers and Reg3a, may also signify a compromised gut barrier function due to inflammation, commonly referred to as 'increased intestinal permeability' or 'leaky gut'.

Reg3a levels positively correlated with calprotectin levels (total S100A8/A9) on days 0 and 3. Interestingly, plasma Reg3a levels increased over time, but calprotectin levels tended to decrease. Further studies on the relationship between these two markers are warranted.

Although not significant, a quick increase in iFABP on day 1 in both groups can indicate mucosal damage after ETEC infection, returning to the baseline level on day 3. A previous human challenge study with ETEC strain H10407 found an increase in plasma iFABP peaking on day 3 in six volunteers with moderate to severe diarrhea and in six volunteers without symptoms [2]. This may be due to the ETEC strain used, as H10407 is regarded as an atypical, virulent ETEC strain with three toxin types and normally causes more severe diarrheal disease than the two strains used in the present study. Instead of iFABP, we identified Reg3a and calprotectin as potential plasma markers that showed changes over time.

Plasma CRP, SAAt, and PAr levels on day 3 showed positive correlations with stool ETEC DNA levels, indicating that higher levels of intestinal ETEC proliferation are associated with increased levels of systemic inflammation.

Our findings support the presence of induced systemic inflammation and mucosal injury during non-invasive ETEC infection, notably marked by an increase in CRP and SAAt with a decreased truncation pattern in SAA1.1 and Reg3a during the early infection phase in the SP group. Inflammatory markers were correlated with mucosal injury markers and maximum ETEC proliferation levels. The plasma concentrations of some kynurenines, namely KTR and Pic, and their correlations with inflammation markers indicate their roles in the inflammatory response during ETEC infection. Plasma PLP is a known cofactor and decreased over the follow-up period in the SP group, inversely correlating with the maximum ETEC proliferation level, which emphasizes the importance of the kynurenine pathway. Reg3a, a mucosal injury marker, increased in the SP group, indicating its potential protective role for the gut mucosa. These findings add to our understanding of how ETEC infection can impact host inflammatory and immune responses and may have implications for long-term negative health outcomes following ETEC infection.

### Limitations

In this study, we only measured biomarkers in plasma, but incorporating measurements of some of these markers in stool samples would have offered more gastrointestinal-specific validation of our findings. In contrast to other studies that used only one strain for experimental infection, we used two strains, TW11681 and TW10722, which might vary somewhat in their ability to elicit mucosal injury and inflammation. However, we did not see a clear difference between the strains' ability to cause inflammation in our data, and the number of volunteers was too small to directly compare the two ETEC strains. We chose to use the maximum proliferation levels for the formation of groups in this study, rather than inoculum dose or diarrheal disease, since gut lumen levels of bacteria vastly outnumber the initial dose when ETEC colonizes.

It is possible that some biomarker actually peaked on days 4, 5, or 6 during the experimental infection phase, when samples were not collected. Also, some of the stools that were passed by the volunteers could not be analyzed as detailed in Vedøy et al*.* [24, 27], with a possibility that we did not capture the absolute maximum ETEC stool concentrations. This may be the reason for poor correlations between plasma biomarkers and ETEC proliferation.

This study was performed on healthy young Norwegian adults, and our results may therefore not be directly comparable to young children living in ETEC endemic areas, where ST-ETEC has been shown to cause severe, sometimes fatal, diarrhea and longer-term stunting [4]. There are several reasons contributing to this higher disease burden and sequels in young children, such as frequent enteric co-pathogens, malnutrition and small bodies more quickly becoming dehydrated [65, 66]. Although data are still scarce on ETEC induced systemic inflammation in children, they may play an important role for disease severity in young children.

## Supplementary Information

Below is the link to the electronic supplementary material.

Supplementary file1 (DOCX 1046 KB)

## Data Availability

Most data from this study are presented in the manuscript and supplementary material. Raw data for the findings of this study were uploaded to a data archiving platform, Sikt (http://www.sikt.no/), in the following location (https://doi.org/10.18712/NSD-NSD3130-V1).
