# Supplementary material for: Inflammation, the kynurenines, and mucosal injury during human experimental enterotoxigenic Escherichia coli infection
Source: Med Microbiol Immunol. 2024 Mar 2;213(1):2. doi: 10.1007/s00430-024-00786-z (PMC10908629; doi:10.1007/s00430-024-00786-z)
Supplement: Supplementary file 1 — Supplementary file1 (DOCX 1046 KB) [file 430_2024_786_MOESM1_ESM.docx]

Supplementary material

**Inflammation, the kynurenines and mucosal injury during human experimental enterotoxigenic *Escherichia coli* infection**

Sehee Rim^1^, Oda Barth Vedøy^1^, Ingeborg Brønstad^2,3^, Adrian McCann^4^, Klaus Meyer^4^, Hans Steinsland^5,6^, Kurt Hanevik^1,7^

1. Department of Clinical Science, Faculty of Medicine, University of Bergen, Bergen, Norway
2. National Centre for Ultrasound in Gastroenterology, Haukeland University Hospital, Bergen, Norway
3. Department of Medicine, Haukeland University Hospital, Bergen, Norway
4. Bevital AS, Bergen, Norway
5. Centre for Intervention Science in Maternal and Child Health, Centre for International Health, Department of Global Public Health and Primary Care, Faculty of Medicine, University of Bergen, Bergen, Norway
6. Department of Biomedicine, Faculty of Medicine, University of Bergen, Bergen, Norway
7. National Center for Tropical Infectious Diseases, Department of Medicine, Haukeland University Hospital, Bergen, Norway

|  | Abbreiviation | Metabolite | Reported Values (min) | Reported Values (max) | SP (N=14) | LP (N=7) |
| --- | --- | --- | --- | --- | --- | --- |
| B1 | **Thi** | Thiamine | 2 | 50 | **0.001** | 0.314 |
|  | **TMP** | Thiamine monophosphate | 2 | 15 | **0.010** | **0.001** |
| B2 | **Ribo** | Riboflavin | 5 | 100 | **0.034** | **0.015** |
|  | **FMN** | Flavin mononucleotide | 3 | 30 | **0.018** | 0.696 |
| B6 | **PLP** | Pyridoxal 5'-phosphate | 15 | 150 | **0.000** | **0.046** |
|  | **PL** | Pyridoxal | 5 | 150 | 0.077 | **0.019** |
|  | **PA** | 4-Pyridoxic acid | 10 | 200 | 0.207 | 0.683 |
|  | **PAr** | PA:(PLP+PL) | 0.29 | 0.51 | 0.092 | 0.060 |
| Kynurenine | **Kyn/Trp** | Kynurenine/tryptophan ratio |  |  | **0.000** | 0.073 |
|  | **Kyn** | Kynurenine | 1.0 | 3.3 | **0.006** | 0.164 |
|  | **Trp** | Tryptophan | 43.0 | 89 | **0.001** | 0.119 |
|  | **KA** | Kynurenic acid | 1 | 2.9 | **0.016** | 0.203 |
|  | **XA** | Xanthurenic acid | 2 | 35 | 0.976 | 0.371 |
|  | **AA** | Anthranilic acid | 7 | 30 | **0.007** | 0.603 |
|  | **HAA** | 3-Hydroxyanthranilic acid | 10 | 80 | **0.042** | 0.454 |
|  | **Pic** | Picolinic acid | 20 | 100 | **0.001** | 0.869 |
|  | **HK** | 3'-hydroxykynurenine | 25 | 80 | 0.602 | **0.036** |
|  | **QA** | Quinolinic acid | 150 | 700 | **0.005** | 0.179 |
|  | **NAM** | Nicotinamide | 100 | 600 | **0.001** | 0.302 |
|  | **mNAM** | N1-methylnicotinamide | 20 | 250 | 0.065 | 0.376 |
| Neopt | **Neopt** | Neopterin | 5 | 50 | **0.000** | 0.142 |
| CRP | **CRP** | C-reactive protein | 0 | 10 | **0.001** | 0.195 |
| Cystatin C | **Cysta** | Cystatin | 0 | 7 | 0.064 | 0.070 |
| Calprotectin | **S100A8** | Calprotectin | 0 | 20 | 0.076 | **0.007** |
|  | **S100A9** | Calprotectin | 0 | 20 | 0.287 | 0.105 |
|  | **S100At** | Calprotectin | 0 | 20 | 0.563 | 0.115 |
| Serum Amyloid A | **SAA1.1** | Serum amyloid A variants | 0 | 20 | **0.001** | **0.032** |
|  | **SAA1.2** | Serum amyloid A variants | 0 | 20 | 0.375 | 0.447 |
|  | **SAA1.3** | Serum amyloid A variants | 0 | 20 | **0.002** | 0.149 |
|  | **SAA2.1** | Serum amyloid A variants | 0 | 20 | **0.000** | 0.152 |
|  | **SAA2.2** | Serum amyloid A variants | 0 | 20 | **0.006** | 0.650 |
|  | **SAAt** | Serum amyloid A variants | 0 | 20 | **0.000** | **0.017** |
| Reg3a | | Regenerating islet-derived protein III-alpha |  |  | **0.000** | 0.849 |
| IFABP | | Intestinal Fatty Acid Binding Protein |  |  | 0.383 | 0.905 |

**Supplementary table 1.** The table displays the p-values resulting from the Friedman's test conducted for each marker across all timepoints. P-values highlighted in **bold** indicate statistically significant results obtained from the Friedman's test with p-value less than 0.05, and p-values highlighted in red indicate statistically significant results obtained from the post-hoc tests with p-value less than 0.05, with bold and red p-values indicating significant p-values from both Friedman and post-hoc tests.

|  |  | Day-0_SP | Day-1_SP | Day-2_SP | Day-3_SP |
| --- | --- | --- | --- | --- | --- |
| AA | **Day-0** | 1 | 1 | 0.105344 | 0.385313 |
|  | **Day-1** | 1 | 1 | 0.827273 | 1 |
|  | **Day-2** | 0.105344 | 0.827273 | 1 | 1 |
|  | **Day-3** | 0.385313 | 1 | 1 | 1 |
|  | **Day-7** | 1 | 1 | 0.040505 | 0.165438 |
| CRP | **Day-0** | 1 | 1 | 0.464706 | 0.041419 |
|  | **Day-1** | 1 | 1 | 0.079713 | 0.004903 |
|  | **Day-2** | 0.464706 | 0.079713 | 1 | 1 |
|  | **Day-3** | 0.041419 | 0.004903 | 1 | 1 |
|  | **Day-7** | 1 | 0.406218 | 1 | 1 |
| FMN | **Day-0** | 1 | 0.561582 | 0.039241 | 0.06401 |
|  | **Day-1** | 0.561582 | 1 | 1 | 1 |
|  | **Day-2** | 0.039241 | 1 | 1 | 1 |
|  | **Day-3** | 0.06401 | 1 | 1 | 1 |
|  | **Day-7** | 1 | 1 | 1 | 1 |
| HAA | **Day-0** | 1 | 1 | 0.505877 | 0.388805 |
|  | **Day-1** | 1 | 1 | 1 | 1 |
|  | **Day-2** | 0.505877 | 1 | 1 | 1 |
|  | **Day-3** | 0.388805 | 1 | 1 | 1 |
|  | **Day-7** | 1 | 1 | 0.296141 | 0.223584 |
| HK | **Day-0** | 1 | 1 | 1 | 1 |
|  | **Day-1** | 1 | 1 | 1 | 1 |
|  | **Day-2** | 1 | 1 | 1 | 1 |
|  | **Day-3** | 1 | 1 | 1 | 1 |
|  | **Day-7** | 1 | 1 | 1 | 1 |
| IFABP | **Day-0** | 1 | 1 | 1 | 1 |
|  | **Day-1** | 1 | 1 | 1 | 1 |
|  | **Day-2** | 1 | 1 | 1 | 1 |
|  | **Day-3** | 1 | 1 | 1 | 1 |
|  | **Day-7** | 1 | 1 | 1 | 1 |
| KA | **Day-0** | 1 | 1 | 0.024927 | 0.124234 |
|  | **Day-1** | 1 | 1 | 1 | 1 |
|  | **Day-2** | 0.024927 | 1 | 1 | 1 |
|  | **Day-3** | 0.124234 | 1 | 1 | 1 |
|  | **Day-7** | 1 | 1 | 0.832862 | 1 |
| Kyn/Trp | **Day-0** | 1 | 1 | 0.024927 | 0.017696 |
|  | **Day-1** | 1 | 1 | 0.296141 | 0.223584 |
|  | **Day-2** | 0.024927 | 0.296141 | 1 | 1 |
|  | **Day-3** | 0.017696 | 0.223584 | 1 | 1 |
|  | **Day-7** | 1 | 1 | 0.048455 | 0.034877 |
| mNAM | **Day-0** | 1 | 1 | 1 | 1 |
|  | **Day-1** | 1 | 1 | 1 | 1 |
|  | **Day-2** | 1 | 1 | 1 | 1 |
|  | **Day-3** | 1 | 1 | 1 | 1 |
|  | **Day-7** | 1 | 1 | 0.124234 | 0.296141 |
| NAM | **Day-0** | 1 | 0.440189 | 0.024503 | 1 |
|  | **Day-1** | 0.440189 | 1 | 1 | 0.570554 |
|  | **Day-2** | 0.024503 | 1 | 1 | 0.034317 |
|  | **Day-3** | 1 | 0.570554 | 0.034317 | 1 |
|  | **Day-7** | 1 | 0.385313 | 0.020652 | 1 |
| Neopt | **Day-0** | 1 | 1 | 0.566066 | 0.000592 |
|  | **Day-1** | 1 | 1 | 1 | 0.03987 |
|  | **Day-2** | 0.566066 | 1 | 1 | 0.189373 |
|  | **Day-3** | 0.000592 | 0.03987 | 0.189373 | 1 |
|  | **Day-7** | 0.821683 | 1 | 1 | 0.121171 |
| PA | **Day-0** | 1 | 1 | 0.221207 | 1 |
|  | **Day-1** | 1 | 1 | 1 | 1 |
|  | **Day-2** | 0.221207 | 1 | 1 | 1 |
|  | **Day-3** | 1 | 1 | 1 | 1 |
|  | **Day-7** | 1 | 1 | 1 | 1 |
| PAr | **Day-0** | 1 | 1 | 1 | 0.124234 |
|  | **Day-1** | 1 | 1 | 1 | 0.652157 |
|  | **Day-2** | 1 | 1 | 1 | 1 |
|  | **Day-3** | 0.124234 | 0.652157 | 1 | 1 |
|  | **Day-7** | 1 | 1 | 1 | 1 |
| PL | **Day-0** | 1 | 1 | 0.647305 | 0.191504 |
|  | **Day-1** | 1 | 1 | 1 | 1 |
|  | **Day-2** | 0.647305 | 1 | 1 | 1 |
|  | **Day-3** | 0.191504 | 1 | 1 | 1 |
|  | **Day-7** | 0.165438 | 1 | 1 | 1 |
| PLP | **Day-0** | 1 | 0.142621 | 0.005968 | 0.000274 |
|  | **Day-1** | 0.142621 | 1 | 1 | 0.440189 |
|  | **Day-2** | 0.005968 | 1 | 1 | 1 |
|  | **Day-3** | 0.000274 | 0.440189 | 1 | 1 |
|  | **Day-7** | 0.014599 | 1 | 1 | 1 |
| Pic | **Day-0** | 1 | 1 | 0.048455 | 0.001361 |
|  | **Day-1** | 1 | 1 | 1 | 0.124234 |
|  | **Day-2** | 0.048455 | 1 | 1 | 1 |
|  | **Day-3** | 0.001361 | 0.124234 | 1 | 1 |
|  | **Day-7** | 0.124234 | 1 | 1 | 1 |
| QA | **Day-0** | 1 | 1 | 1 | 0.436384 |
|  | **Day-1** | 1 | 1 | 0.49759 | 0.004862 |
|  | **Day-2** | 1 | 0.49759 | 1 | 0.925911 |
|  | **Day-3** | 0.436384 | 0.004862 | 0.925911 | 1 |
|  | **Day-7** | 1 | 0.218841 | 1 | 1 |
| Reg3a | **Day-0** | 1 | 1 | 0.16736 | 0.001995 |
|  | **Day-1** | 1 | 1 | 1 | 0.034877 |
|  | **Day-2** | 0.16736 | 1 | 1 | 1 |
|  | **Day-3** | 0.001995 | 0.034877 | 1 | 1 |
|  | **Day-7** | 0.024927 | 0.296141 | 1 | 1 |
| Ribo | **Day-0** | 1 | 1 | 1 | 0.931877 |
|  | **Day-1** | 1 | 1 | 1 | 1 |
|  | **Day-2** | 1 | 1 | 1 | 1 |
|  | **Day-3** | 0.931877 | 1 | 1 | 1 |
|  | **Day-7** | 1 | 0.647305 | 0.191504 | 0.065883 |
| S100A8 | **Day-0** | 1 | 1 | 1 | 1 |
|  | **Day-1** | 1 | 1 | 1 | 1 |
|  | **Day-2** | 1 | 1 | 1 | 1 |
|  | **Day-3** | 1 | 1 | 1 | 1 |
|  | **Day-7** | 0.06683 | 1 | 1 | 1 |
| S100A9 | **Day-0** | 1 | 1 | 1 | 1 |
|  | **Day-1** | 1 | 1 | 1 | 0.647305 |
|  | **Day-2** | 1 | 1 | 1 | 1 |
|  | **Day-3** | 1 | 0.647305 | 1 | 1 |
|  | **Day-7** | 1 | 1 | 1 | 1 |
| S100At | **Day-0** | 1 | 1 | 1 | 1 |
|  | **Day-1** | 1 | 1 | 1 | 1 |
|  | **Day-2** | 1 | 1 | 1 | 1 |
|  | **Day-3** | 1 | 1 | 1 | 1 |
|  | **Day-7** | 1 | 1 | 1 | 1 |
| SAA1.1 | **Day-0** | 1 | 1 | 0.091476 | 0.004223 |
|  | **Day-1** | 1 | 1 | 0.505877 | 0.034877 |
|  | **Day-2** | 0.091476 | 0.505877 | 1 | 1 |
|  | **Day-3** | 0.004223 | 0.034877 | 1 | 1 |
|  | **Day-7** | 1 | 1 | 1 | 0.388805 |
| SAA1.2 | **Day-0** | 1 | 1 | 1 | 1 |
|  | **Day-1** | 1 | 1 | 1 | 1 |
|  | **Day-2** | 1 | 1 | 1 | 1 |
|  | **Day-3** | 1 | 1 | 1 | 1 |
|  | **Day-7** | 1 | 0.884106 | 1 | 1 |
| SAA1.3 | **Day-0** | 1 | 1 | 0.16736 | 0.008748 |
|  | **Day-1** | 1 | 1 | 0.652157 | 0.048455 |
|  | **Day-2** | 0.16736 | 0.652157 | 1 | 1 |
|  | **Day-3** | 0.008748 | 0.048455 | 1 | 1 |
|  | **Day-7** | 1 | 1 | 1 | 0.16736 |
| SAA2.1 | **Day-0** | 1 | 1 | 0.012481 | 0.000925 |
|  | **Day-1** | 1 | 1 | 0.223584 | 0.024927 |
|  | **Day-2** | 0.012481 | 0.223584 | 1 | 1 |
|  | **Day-3** | 0.000925 | 0.024927 | 1 | 1 |
|  | **Day-7** | 0.652157 | 1 | 1 | 0.223584 |
| SAA2.2 | **Day-0** | 1 | 1 | 0.505877 | 0.034877 |
|  | **Day-1** | 1 | 1 | 0.652157 | 0.048455 |
|  | **Day-2** | 0.505877 | 0.652157 | 1 | 1 |
|  | **Day-3** | 0.034877 | 0.048455 | 1 | 1 |
|  | **Day-7** | 0.652157 | 0.832862 | 1 | 1 |
| SAAt | **Day-0** | 1 | 1 | 0.06683 | 0.000925 |
|  | **Day-1** | 1 | 1 | 1 | 0.034877 |
|  | **Day-2** | 0.06683 | 1 | 1 | 1 |
|  | **Day-3** | 0.000925 | 0.034877 | 1 | 1 |
|  | **Day-7** | 0.223584 | 1 | 1 | 0.652157 |
| TMP | **Day-0** | 1 | 0.827273 | 1 | 1 |
|  | **Day-1** | 0.827273 | 1 | 0.122698 | 1 |
|  | **Day-2** | 1 | 0.122698 | 1 | 0.931877 |
|  | **Day-3** | 1 | 1 | 0.931877 | 1 |
|  | **Day-7** | 0.254968 | 1 | 0.029023 | 1 |
| Thi | **Day-0** | 1 | 1 | 1 | 0.012481 |
|  | **Day-1** | 1 | 1 | 1 | 0.832862 |
|  | **Day-2** | 1 | 1 | 1 | 0.034877 |
|  | **Day-3** | 0.012481 | 0.832862 | 0.034877 | 1 |
|  | **Day-7** | 0.06683 | 1 | 0.16736 | 1 |
| XA | **Day-0** | 1 | 1 | 1 | 1 |
|  | **Day-1** | 1 | 1 | 1 | 1 |
|  | **Day-2** | 1 | 1 | 1 | 1 |
|  | **Day-3** | 1 | 1 | 1 | 1 |
|  | **Day-7** | 1 | 1 | 1 | 1 |
| Trp | **Day-0** | 1 | 1 | 0.142621 | 0.827273 |
|  | **Day-1** | 1 | 1 | 1 | 1 |
|  | **Day-2** | 0.142621 | 1 | 1 | 1 |
|  | **Day-3** | 0.827273 | 1 | 1 | 1 |
|  | **Day-7** | 1 | 0.105344 | 0.002355 | 0.024503 |
| Kyn | **Day-0** | 1 | 1 | 0.388805 | 0.06683 |
|  | **Day-1** | 1 | 1 | 0.223584 | 0.034877 |
|  | **Day-2** | 0.388805 | 0.223584 | 1 | 1 |
|  | **Day-3** | 0.06683 | 0.034877 | 1 | 1 |
|  | **Day-7** | 1 | 1 | 1 | 1 |
| Cysta | **Day-0** | 1 | 0.570554 | 1 | 1 |
|  | **Day-1** | 0.570554 | 1 | 0.570554 | 1 |
|  | **Day-2** | 1 | 0.570554 | 1 | 1 |
|  | **Day-3** | 1 | 1 | 1 | 1 |
|  | **Day-7** | 1 | 0.065883 | 1 | 1 |

**Supplementary table 2-1.** The table displays the p-values derived from post-hoc tests, which were conducted to discern time points with statistically significant differences within the SP group. The tests compared the day on the x-axis with the day on the y-axis. Highlighted values in red in the table represent statistically significant results (p < 0.05) indicating a difference between the timepoint on the x-axis and the timepoint on the y-axis, and the values with 1 indicate non-significance.

|  |  | Day-0_LP | Day-1_LP | Day-2_LP | Day-3_LP | Day-7_LP |
| --- | --- | --- | --- | --- | --- | --- |
| AA | **Day-0** | 1 | 1 | 1 | 1 | 1 |
|  | **Day-1** | 1 | 1 | 1 | 1 | 1 |
|  | **Day-2** | 1 | 1 | 1 | 1 | 1 |
|  | **Day-3** | 1 | 1 | 1 | 1 | 1 |
|  | **Day-7** | 1 | 1 | 1 | 1 | 1 |
| CRP | **Day-0** | 1 | 1 | 1 | 0.310249 | 1 |
|  | **Day-1** | 1 | 1 | 1 | 1 | 1 |
|  | **Day-2** | 1 | 1 | 1 | 1 | 1 |
|  | **Day-3** | 0.310249 | 1 | 1 | 1 | 1 |
|  | **Day-7** | 1 | 1 | 1 | 1 | 1 |
| FMN | **Day-0** | 1 | 1 | 1 | 1 | 1 |
|  | **Day-1** | 1 | 1 | 1 | 1 | 1 |
|  | **Day-2** | 1 | 1 | 1 | 1 | 1 |
|  | **Day-3** | 1 | 1 | 1 | 1 | 1 |
|  | **Day-7** | 1 | 1 | 1 | 1 | 1 |
| HAA | **Day-0** | 1 | 1 | 1 | 1 | 0.843966 |
|  | **Day-1** | 1 | 1 | 1 | 1 | 1 |
|  | **Day-2** | 1 | 1 | 1 | 1 | 1 |
|  | **Day-3** | 1 | 1 | 1 | 1 | 1 |
|  | **Day-7** | 0.843966 | 1 | 1 | 1 | 1 |
| HK | **Day-0** | 1 | 1 | 1 | 1 | 0.070537 |
|  | **Day-1** | 1 | 1 | 1 | 1 | 0.310249 |
|  | **Day-2** | 1 | 1 | 1 | 1 | 0.612189 |
|  | **Day-3** | 1 | 1 | 1 | 1 | 0.843966 |
|  | **Day-7** | 0.070537 | 0.310249 | 0.612189 | 0.843966 | 1 |
| IFABP | **Day-0** | 1 | 1 | 1 | 1 | 1 |
|  | **Day-1** | 1 | 1 | 1 | 1 | 1 |
|  | **Day-2** | 1 | 1 | 1 | 1 | 1 |
|  | **Day-3** | 1 | 1 | 1 | 1 | 1 |
|  | **Day-7** | 1 | 1 | 1 | 1 | 1 |
| KA | **Day-0** | 1 | 1 | 1 | 1 | 1 |
|  | **Day-1** | 1 | 1 | 1 | 1 | 0.438399 |
|  | **Day-2** | 1 | 1 | 1 | 1 | 1 |
|  | **Day-3** | 1 | 1 | 1 | 1 | 1 |
|  | **Day-7** | 1 | 0.438399 | 1 | 1 | 1 |
| Kyn/Trp | **Day-0** | 1 | 1 | 1 | 1 | 1 |
|  | **Day-1** | 1 | 1 | 1 | 1 | 1 |
|  | **Day-2** | 1 | 1 | 1 | 1 | 0.103459 |
|  | **Day-3** | 1 | 1 | 1 | 1 | 0.843966 |
|  | **Day-7** | 1 | 1 | 0.103459 | 0.843966 | 1 |
| mNAM | **Day-0** | 1 | 1 | 1 | 1 | 1 |
|  | **Day-1** | 1 | 1 | 1 | 1 | 1 |
|  | **Day-2** | 1 | 1 | 1 | 1 | 0.612189 |
|  | **Day-3** | 1 | 1 | 1 | 1 | 1 |
|  | **Day-7** | 1 | 1 | 0.612189 | 1 | 1 |
| NAM | **Day-0** | 1 | 0.832567 | 1 | 1 | 1 |
|  | **Day-1** | 0.832567 | 1 | 1 | 1 | 1 |
|  | **Day-2** | 1 | 1 | 1 | 1 | 1 |
|  | **Day-3** | 1 | 1 | 1 | 1 | 1 |
|  | **Day-7** | 1 | 1 | 1 | 1 | 1 |
| Neopt | **Day-0** | 1 | 1 | 1 | 1 | 0.304305 |
|  | **Day-1** | 1 | 1 | 1 | 1 | 1 |
|  | **Day-2** | 1 | 1 | 1 | 1 | 1 |
|  | **Day-3** | 1 | 1 | 1 | 1 | 0.973443 |
|  | **Day-7** | 0.304305 | 1 | 1 | 0.973443 | 1 |
| PA | **Day-0** | 1 | 1 | 1 | 1 | 1 |
|  | **Day-1** | 1 | 1 | 1 | 1 | 1 |
|  | **Day-2** | 1 | 1 | 1 | 1 | 1 |
|  | **Day-3** | 1 | 1 | 1 | 1 | 1 |
|  | **Day-7** | 1 | 1 | 1 | 1 | 1 |
| PAr | **Day-0** | 1 | 1 | 1 | 1 | 1 |
|  | **Day-1** | 1 | 1 | 1 | 0.103459 | 1 |
|  | **Day-2** | 1 | 1 | 1 | 0.612189 | 1 |
|  | **Day-3** | 1 | 0.103459 | 0.612189 | 1 | 1 |
|  | **Day-7** | 1 | 1 | 1 | 1 | 1 |
| PL | **Day-0** | 1 | 1 | 1 | 1 | 0.04502 |
|  | **Day-1** | 1 | 1 | 1 | 1 | 0.298397 |
|  | **Day-2** | 1 | 1 | 1 | 1 | 0.249492 |
|  | **Day-3** | 1 | 1 | 1 | 1 | 0.501984 |
|  | **Day-7** | 0.04502 | 0.298397 | 0.249492 | 0.501984 | 1 |
| PLP | **Day-0** | 1 | 0.602795 | 0.709612 | 1 | 0.121934 |
|  | **Day-1** | 0.602795 | 1 | 1 | 1 | 1 |
|  | **Day-2** | 0.709612 | 1 | 1 | 1 | 1 |
|  | **Day-3** | 1 | 1 | 1 | 1 | 0.709612 |
|  | **Day-7** | 0.121934 | 1 | 1 | 0.709612 | 1 |
| Pic | **Day-0** | 1 | 1 | 1 | 1 | 1 |
|  | **Day-1** | 1 | 1 | 1 | 1 | 1 |
|  | **Day-2** | 1 | 1 | 1 | 1 | 1 |
|  | **Day-3** | 1 | 1 | 1 | 1 | 1 |
|  | **Day-7** | 1 | 1 | 1 | 1 | 1 |
| QA | **Day-0** | 1 | 1 | 1 | 1 | 1 |
|  | **Day-1** | 1 | 1 | 0.843966 | 1 | 0.310249 |
|  | **Day-2** | 1 | 0.843966 | 1 | 1 | 1 |
|  | **Day-3** | 1 | 1 | 1 | 1 | 1 |
|  | **Day-7** | 1 | 0.310249 | 1 | 1 | 1 |
| Reg3a | **Day-0** | 1 | 1 | 1 | 1 | 1 |
|  | **Day-1** | 1 | 1 | 1 | 1 | 1 |
|  | **Day-2** | 1 | 1 | 1 | 1 | 1 |
|  | **Day-3** | 1 | 1 | 1 | 1 | 1 |
|  | **Day-7** | 1 | 1 | 1 | 1 | 1 |
| Ribo | **Day-0** | 1 | 1 | 1 | 1 | 0.510408 |
|  | **Day-1** | 1 | 1 | 1 | 1 | 0.031155 |
|  | **Day-2** | 1 | 1 | 1 | 1 | 0.430844 |
|  | **Day-3** | 1 | 1 | 1 | 1 | 0.254688 |
|  | **Day-7** | 0.510408 | 0.031155 | 0.430844 | 0.254688 | 1 |
| S100A8 | **Day-0** | 1 | 0.032147 | 1 | 0.103459 | 0.310249 |
|  | **Day-1** | 0.032147 | 1 | 0.843966 | 1 | 1 |
|  | **Day-2** | 1 | 0.843966 | 1 | 1 | 1 |
|  | **Day-3** | 0.103459 | 1 | 1 | 1 | 1 |
|  | **Day-7** | 0.310249 | 1 | 1 | 1 | 1 |
| S100A9 | **Day-0** | 1 | 0.612189 | 1 | 0.217194 | 1 |
|  | **Day-1** | 0.612189 | 1 | 1 | 1 | 1 |
|  | **Day-2** | 1 | 1 | 1 | 1 | 1 |
|  | **Day-3** | 0.217194 | 1 | 1 | 1 | 1 |
|  | **Day-7** | 1 | 1 | 1 | 1 | 1 |
| S100At | **Day-0** | 1 | 0.310249 | 1 | 0.438399 | 0.843966 |
|  | **Day-1** | 0.310249 | 1 | 1 | 1 | 1 |
|  | **Day-2** | 1 | 1 | 1 | 1 | 1 |
|  | **Day-3** | 0.438399 | 1 | 1 | 1 | 1 |
|  | **Day-7** | 0.843966 | 1 | 1 | 1 | 1 |
| SAA1.1 | **Day-0** | 1 | 0.709612 | 1 | 1 | 1 |
|  | **Day-1** | 0.709612 | 1 | 1 | 1 | 0.083281 |
|  | **Day-2** | 1 | 1 | 1 | 1 | 1 |
|  | **Day-3** | 1 | 1 | 1 | 1 | 0.362604 |
|  | **Day-7** | 1 | 0.083281 | 1 | 0.362604 | 1 |
| SAA1.2 | **Day-0** | 1 | 1 | 1 | 1 | 1 |
|  | **Day-1** | 1 | 1 | 1 | 1 | 1 |
|  | **Day-2** | 1 | 1 | 1 | 1 | 1 |
|  | **Day-3** | 1 | 1 | 1 | 1 | 1 |
|  | **Day-7** | 1 | 1 | 1 | 1 | 1 |
| SAA1.3 | **Day-0** | 1 | 1 | 1 | 1 | 1 |
|  | **Day-1** | 1 | 1 | 1 | 1 | 0.973443 |
|  | **Day-2** | 1 | 1 | 1 | 1 | 1 |
|  | **Day-3** | 1 | 1 | 1 | 1 | 0.254688 |
|  | **Day-7** | 1 | 0.973443 | 1 | 0.254688 | 1 |
| SAA2.1 | **Day-0** | 1 | 1 | 1 | 1 | 1 |
|  | **Day-1** | 1 | 1 | 1 | 1 | 0.709612 |
|  | **Day-2** | 1 | 1 | 1 | 1 | 1 |
|  | **Day-3** | 1 | 1 | 1 | 1 | 0.510408 |
|  | **Day-7** | 1 | 0.709612 | 1 | 0.510408 | 1 |
| SAA2.2 | **Day-0** | 1 | 1 | 1 | 1 | 1 |
|  | **Day-1** | 1 | 1 | 1 | 1 | 1 |
|  | **Day-2** | 1 | 1 | 1 | 1 | 1 |
|  | **Day-3** | 1 | 1 | 1 | 1 | 1 |
|  | **Day-7** | 1 | 1 | 1 | 1 | 1 |
| SAAt | **Day-0** | 1 | 1 | 1 | 0.973443 | 1 |
|  | **Day-1** | 1 | 1 | 1 | 1 | 0.121934 |
|  | **Day-2** | 1 | 1 | 1 | 1 | 1 |
|  | **Day-3** | 0.973443 | 1 | 1 | 1 | 0.083281 |
|  | **Day-7** | 1 | 0.121934 | 1 | 0.083281 | 1 |
| TMP | **Day-0** | 1 | 0.973443 | 1 | 0.068633 | 0.016995 |
|  | **Day-1** | 0.973443 | 1 | 1 | 1 | 0.832567 |
|  | **Day-2** | 1 | 1 | 1 | 0.430844 | 0.121934 |
|  | **Day-3** | 0.068633 | 1 | 0.430844 | 1 | 1 |
|  | **Day-7** | 0.016995 | 0.832567 | 0.121934 | 1 | 1 |
| Thi | **Day-0** | 1 | 1 | 1 | 0.973443 | 1 |
|  | **Day-1** | 1 | 1 | 1 | 1 | 1 |
|  | **Day-2** | 1 | 1 | 1 | 1 | 1 |
|  | **Day-3** | 0.973443 | 1 | 1 | 1 | 1 |
|  | **Day-7** | 1 | 1 | 1 | 1 | 1 |
| XA | **Day-0** | 1 | 0.540553 | 1 | 1 | 1 |
|  | **Day-1** | 0.540553 | 1 | 1 | 1 | 1 |
|  | **Day-2** | 1 | 1 | 1 | 1 | 1 |
|  | **Day-3** | 1 | 1 | 1 | 1 | 1 |
|  | **Day-7** | 1 | 1 | 1 | 1 | 1 |
| Trp | **Day-0** | 1 | 1 | 1 | 1 | 0.973443 |
|  | **Day-1** | 1 | 1 | 1 | 1 | 0.304305 |
|  | **Day-2** | 1 | 1 | 1 | 1 | 0.832567 |
|  | **Day-3** | 1 | 1 | 1 | 1 | 1 |
|  | **Day-7** | 0.973443 | 0.304305 | 0.832567 | 1 | 1 |
| Kyn | **Day-0** | 1 | 1 | 1 | 1 | 0.843966 |
|  | **Day-1** | 1 | 1 | 1 | 1 | 0.612189 |
|  | **Day-2** | 1 | 1 | 1 | 1 | 1 |
|  | **Day-3** | 1 | 1 | 1 | 1 | 1 |
|  | **Day-7** | 0.843966 | 0.612189 | 1 | 1 | 1 |
| Cysta | **Day-0** | 1 | 1 | 1 | 1 | 1 |
|  | **Day-1** | 1 | 1 | 0.21261 | 0.973443 | 0.21261 |
|  | **Day-2** | 1 | 0.21261 | 1 | 1 | 1 |
|  | **Day-3** | 1 | 0.973443 | 1 | 1 | 1 |
|  | **Day-7** | 1 | 0.21261 | 1 | 1 | 1 |

**Supplementary table 2-2.** The table displays the p-values derived from post-hoc tests, which were conducted to discern time points with statistically significant differences within the LP group. The tests compared the day on the x-axis with the day on the y-axis. Highlighted values in red in the table represent statistically significant results (p < 0.05) indicating a difference between the timepoint on the x-axis and the timepoint on the y-axis, and the values with 1 indicate non-significance.


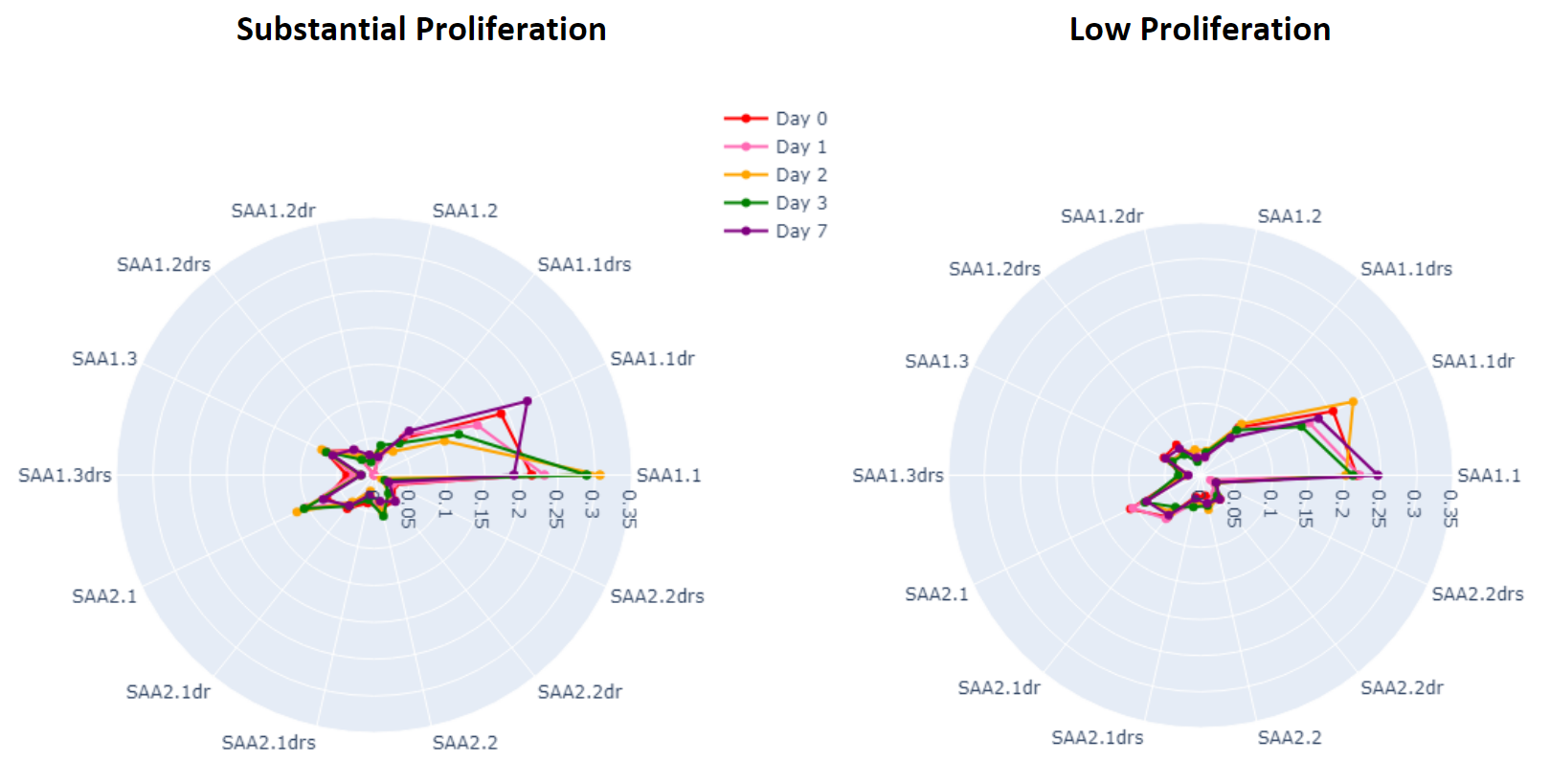


**Supplementary figure 1.** Radar plots describing truncation patterns for SAA isoform ratios over infection phase.


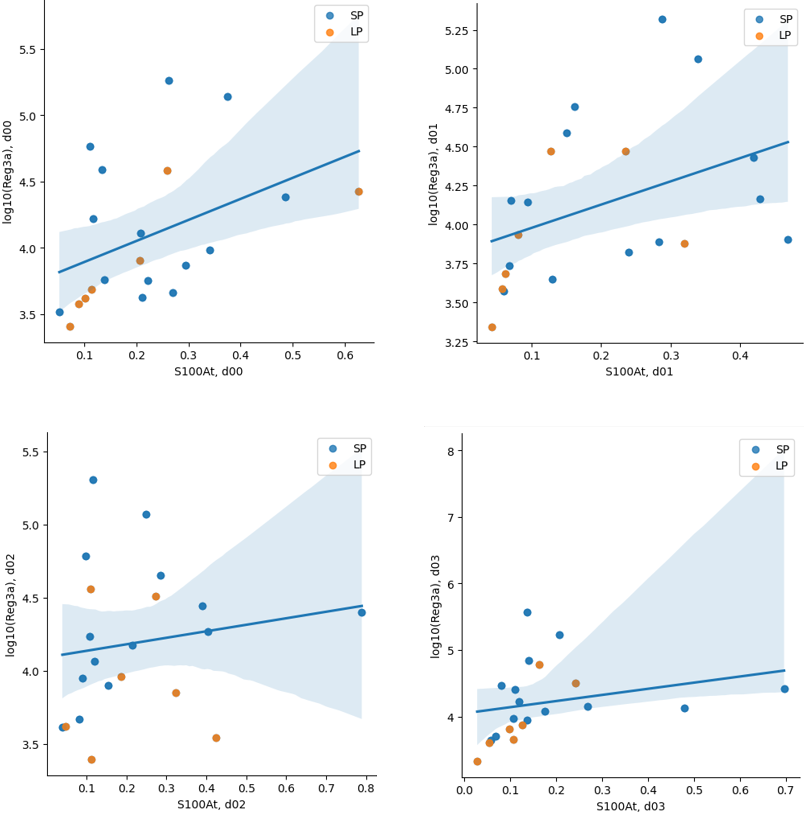


**Supplementary figure 2.** Regression plot for calprotectin (S100At) and log values of Reg3a from baseline to day 3, with base 10. Blue spots represent the SP group, orange spots represent the LP group. This reflects partially the results of Spearman test.

**Supplementary table 3.** Correlations between inflammation markers on day 3 with tryptophan pathway metabolites. Rho- and p-values from spearman tests are listed. (NS=Not significant).

|  | Kyn/trp day 3 | | CRP day 3 | | Neopterin day 3 | | PAr index day 3 | | SAAt day 3 | |
| --- | --- | --- | --- | --- | --- | --- | --- | --- | --- | --- |
|  | **rho** | **p-value** | **rho** | **p-value** | **rho** | **p-value** | **rho** | **p-value** | **rho** | **p-value** |
| AA day 2 | 0.62 | 0.001 | 0.62 | 0.003 | 0.46 | 0.035 | NS | NS | 0.53 | 0.014 |
| HK day 3 | 0.53 | 0.003 | 0.53 | 0.014 | 0.51 | 0.019 | 0.58 | 0.006 | 0.48 | 0.028 |
| QA day 3 | 0.59 | 0.002 | 0.66 | 0.001 | 0.74 | <0.001 | 0.59 | 0.005 | 0.58 | 0.006 |


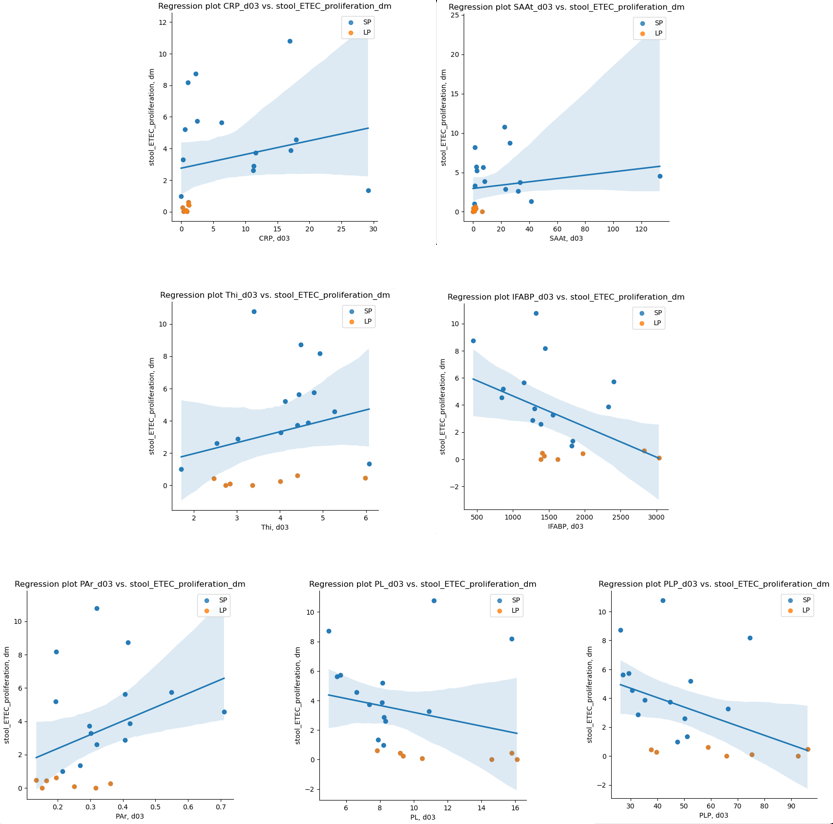


**Supplementary figure 3.** Regression plots showing linear correlation between most prominent correlations between proliferation levels and CRP, SAAt, Thi, PAr, iFABP, PL and PLP on day 3. Linear regression can partially visualize the correlations from Spearman tests.


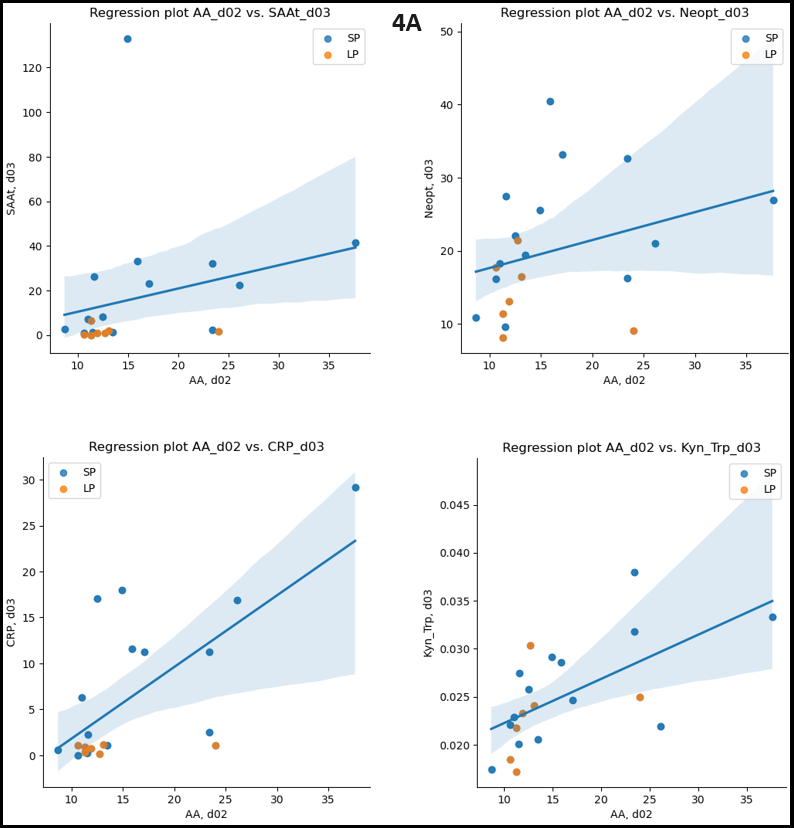


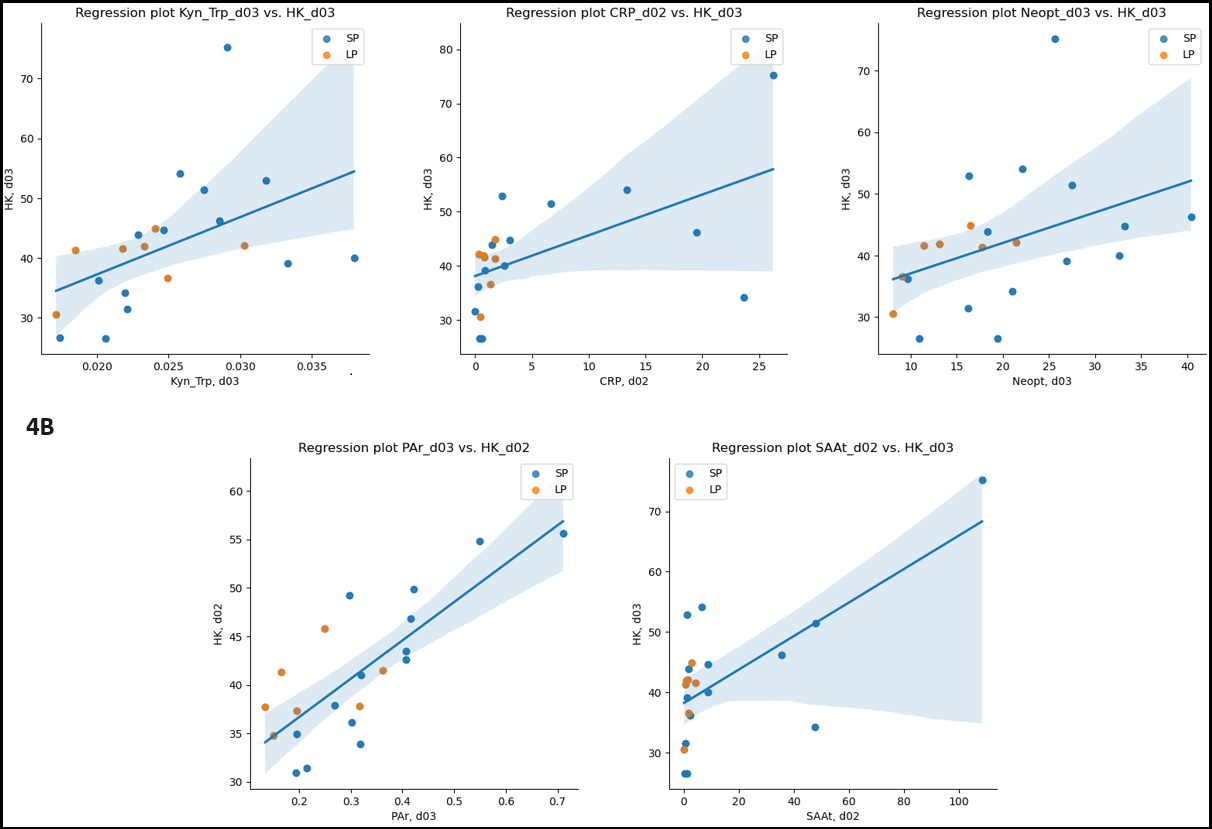

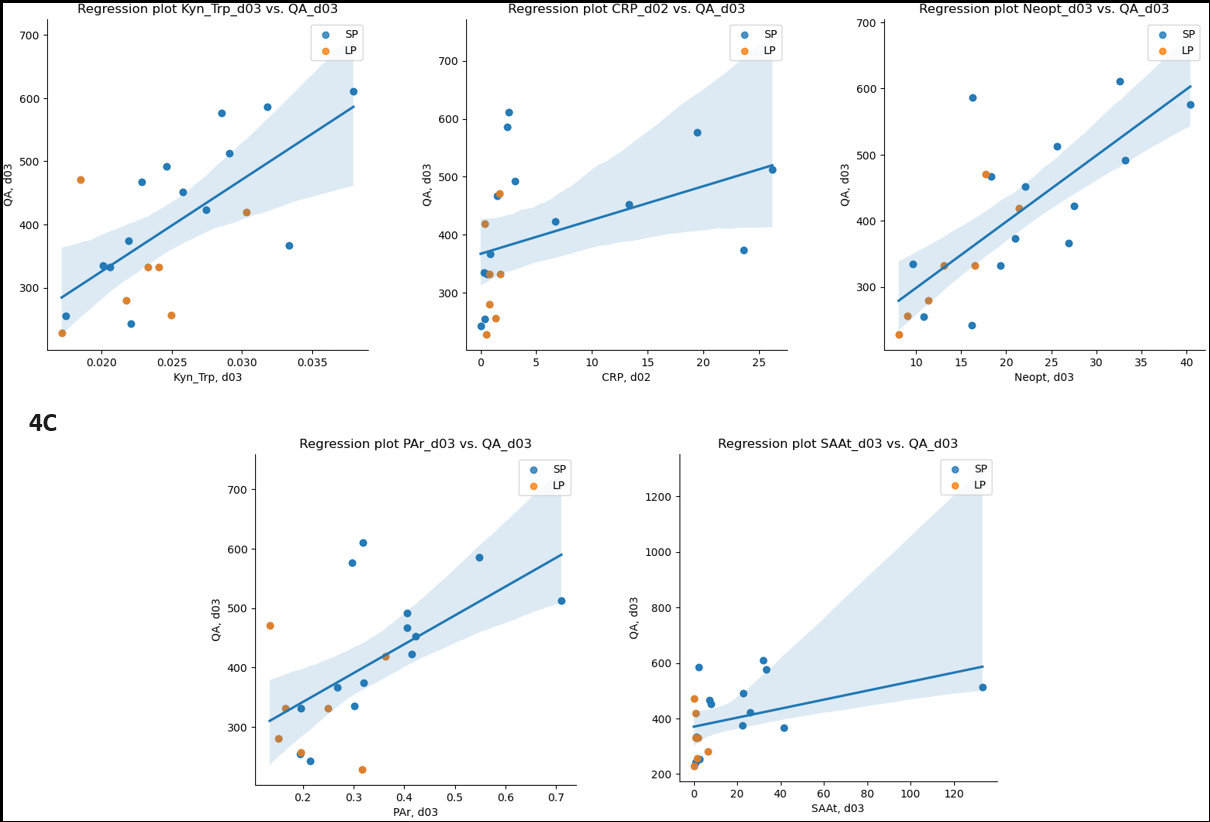


**Supplementary figure 4.** Regression plots showing linear correlation between most prominent correlations between kynurenine metabolites and inflammation markers on day 2, 3. Linear regression can partially visualize the correlations from Spearman tests. Each figure shows correlations between inflammation markers and: A AA; B HK; and C QA.
